# The use and potential abuse of psychoactive plants in southern Africa: an overview of evidence and future potential

**DOI:** 10.3389/fphar.2024.1269247

**Published:** 2024-05-24

**Authors:** Norman Zimunda Nyazema, Jonathan Tinotenda Chanyandura, Bronwyn Egan

**Affiliations:** ^1^ Department of Pharmacy, University of Limpopo, Mankgweng, South Africa; ^2^ Botany Department, University of Limpopo, Mankgweng, South Africa

**Keywords:** psychoactive ethnomedicinal plants, use and potential abuse, harm reduction, traditional medical practitioners, socio-pharmacological study

## Abstract

**Background:**

Most Bantu ethnic groups in southern Africa utilize indigenous herbal medicines, some of which have psychoactive properties. Traditional medical practitioners (TMPs) commonly use them not only for divinatory purposes but to treat and manage mental and other illnesses. Unfortunately, the research on their results, risks, and benefits do not align. Little is known about their potential abuse among TMPs and community members in southern Africa. Herbal medicines are complex because whole plants are sometimes used, unlike in other treatments which use only one active ingredient. However, if the key mechanisms of action of these ethnomedicinal plants can be identified through socio-pharmacological research, useful botanical agents can be developed. A review of socio-pharmacological studies to evaluate the consequences of exposure to ethnomedicinal plants with psychoactive properties was conducted with the aim of identifying harm reduction strategies and investigating how the plants could be developed into useful botanicals.

**Method:**

The search methods involved retrieval of records from PubMed/MEDLINE, Embase, Web of Science, Dissertations and Theses Global, and OpenGrey. The English language and human subjects were used as filters. In addition, some information was obtained from TMPs and community members.

**Results:**

The following psychoactive plants were found to be commonly used or abused: *Boophone disticha, Cannabis sativa, Datura stramonium, Leonotis leonurus, Psilocybe cubensis*, and *Sceletium tortuosum.* The commercialization of *Cannabis, L. leonurus, S. tortuosum,* and *Aspalathus* is growing fast. The abuse liability of *B. disticha, D. stramonium*, *and P. cubensis* appears not to be appreciated. Five countries were found to have TMP policies and three with TMP Councils.

**Conclusion:**

TMPs in the region are aware of the CNS effects of the identified psychoactive plants which can be explored further to develop therapeutic agents. There is a need to work closely with TMPs to reduce harm from the abuse of these plants.

## Introduction

In southern Africa and in the rest of the continent, the COVID-19 pandemic has intensified the struggle around scarce health resources. Previously, more than 80% of the Bantu population in southern Africa had always relied on the use of indigenous herbal medicines, some of which have psychoactive properties. Generally, plants with psychoactive or psychotropic properties, have always been used by traditional medical practitioners (TMPs) for divinatory purposes, treatment, and to manage illness.

TMPs consult the spiritual realm by invoking and conferring with deceased family members. Appropriate action including the prescription of plant medicines and rituals is the prerogative of diviners following the discovery of the cause of any misfortune. In southern African divination, the diviner goes into a trance and appears to be in an altered state of consciousness to assist in the healing. In addition, the diviner is said in that state to be able to communicate with “spirits” and undertake “soul journeys” while dreaming or in a trance. This involves either ceremonially ingested, sniffed, or smoked psychotropic substances (Hewson, 1998). According to the World Health Organization (WHO) definition of traditional medicine, this is to a certain extent not explicable.

WHO (WHO, 2023) defines “traditional medicine” as follows:

The sum total of the knowledge, skill, and practices based on theories, beliefs, and experiences indigenous to different cultures, whether explicable or not, used in the maintenance of health as well as in the prevention, diagnosis, improvement or treatment of physical and mental illness (WHO, 2023).

It can be argued that the pharmacology of some animal, plant, and soil products used in TM can be explained scientifically. This includes plants with psychoactive properties, including those which cross the blood–brain barrier that result in psychotropic effects of interference with brain functions, mood awareness, thoughts, and feelings. These are classified as psychotropic drugs.

The therapies used in traditional medical practice have complex interactions. Treatment with traditional medicine does not involve a single specific therapeutic ingredient but often includes specific diagnostic settings and interaction processes (Hewson, 1998; Mhame et al., 2014). Unfortunately, the research evidence and the possible harm and advantages of these therapies following the use of psychoactive substances among TMPs do not align. For example, little has been reported about potential psychoactive plant abuse liability by TMPs and others in southern Africa. Herbal medicines are complex, and their complexity comes from the fact that whole plants are sometimes used, unlike other treatments that use only one active ingredient . However, if the key mechanisms of action of these ethnomedicinal plants can be identified through socio-pharmacological research, useful botanical agents can be developed.

Social pharmacology, or socio-pharmacology, is a relatively new field in clinical pharmacology. In other words, it describes the relationships between society and drugs whether of ethno-origin or not. The discipline studies the life cycle of any drug used in society. The discipline continues evolving and is currently underappreciated. According to Papadopulos et al. (2021), the societal aspects of therapeutics are more than imperative today, given determinants such as the healthcare system, the political setting, unemployment, and exposure to chronic disease.

There has been very little research into the use of psychoactive plants with psychotropic compounds, whether for divinatory or therapeutic purposes, in southern Africa and Africa in general (Jean-Francois, 2020). Existing reports have always been part of other ethnobotanical investigations. In fact, most studies since 1983 have concentrated on medicinal plants. This can be explained by the bias of 19th century researchers regarding psychoactive plants as part of divinatory and healing practices. At the time, the practices were regarded as witchcraft or primitive, resulting in the neglect of the importance of psychoactive medicinal plants. There has additionally been a loss of the oral transmission of information regarding the use of plants because people have forgotten traditional practices. Nevertheless, there has been some interest in psychoactive plant use in southern Africa (Mitchell and Hudson, 2004; Sobiecki, 2008).

This study reviews the relevant literature on the relationship between society and commonly used plants of ethno-origin that have psychoactive properties that result in psychotropic action. It aims to identify harm reduction strategies and investigate how the plants could be developed into useful botanicals for treating and managing mental illnesses.

## Methods

Studies on the use of psychoactive plants in traditional medical practice in southern Africa were searched from electric databases including PubMed/MEDLINE, Embase, Web of Science, Dissertations and Theses Global, and OpenGrey. The studies were retrieved mainly by applying the English language and, where necessary, the most spoken vernacular Bantu language in the southern African country. The following search terms were used: “divination,” “traditional medical practice,” “psychoactive plants,” “psychotropic plants,” and “witchcraft.” In order to construct a global and relevant picture for a regional perspective, some searches included studies at a sub-Saharan Africa level. Finally, a snowball search was expanded to include references cited in the search publications in order to expand the relevant literature.

The inclusion criteria for the search were anything regarding divination, diagnosis and treatment in traditional medical practice using plants, and the use of these plants in the general community for other purposes. Anything else was excluded in the search.

All plants that were eventually described in the study were authenticated by a botanist and curator at a university in South Africa.

The literature study was then followed by interviews with 15 purposively selected TMPs from five Southern African Development Community (SADC) countries who were willing to provide information on their practices. Some community members were also interviewed about their knowledge of psychoactive plants.

## Results and discussion

Information obtained from the interviewed TMPs seemed to suggest that only five countries in southern Africa had traditional medicine practice policies and three had TMP councils. A close analysis of the functions of these councils showed that they were not that effective in regulating how psychoactive plants were generally used (Table 1).

**TABLE 1:** Plant species, part of plant used, psychotropic constituents, and classification of pharmacological classification.

Plant species	Part of plant used	Psychotropic constituent(s)	Classification of constituents
*Boophone disticha*	Bulb	Amaryllidaceae alkaloids	Hallucinogens
*Cannabis sativa, L*	Flower and leaves	THC	Stimulant/sedative
*Datura stramonium L*	Seeds	Scopolamine and hyoscyamine	Deliriant/hallucinogen
*Helichrysum odoratissimum L*	Whole plant	Decaffeocylquinic acid	Sedative and relaxant
*Leonotis leonurus (L) R.Br*	Leaves and seeds	Adrenocyl–EA and anandamide	Sedative and calamative
*Psilocybe cubensis*	Whole plant	Pscylocybin and pscilocin	Psychedelic and entheogenic
*Mesebryanthemum tortuosum (L)* NE Brown	Leaves and flowers	Mesembrenone and mesembrine	Mood altering
*Silene capensis*	Whole plant	Phytoecdysteroid	Hallucinogen
*Dioscorea dregeana* (Kunth) Durand & Schinz	Tuber	Dioscorine and crinamine	Sedative

However, one of the psychoactive plants, *Cannabis sativa,* is strictly speaking not indigenous but has, over the years since its introduction into southern Africa, found its place in traditional medical practice. *C. sativa* is in the statute books of SADC countries, and efforts to control its abuse are ongoing. With its increased decriminalization and legalization, worldwide harm reduction strategies are being developed. These are very similar to the well-known strategies developed for *Nicotiana tabacum*. *C. sativa*, like the other the psychoactive plants, can be used both for recreational and medicinal purposes. In modern medicine, many chemical substances such as morphine, pseudoephedrine, and alcohol are abused, and those responsible for making them accessible are held accountable for the drug abuse. This, unfortunately, is not currently possible with TMPs whose councils are not that powerful. Moreover, the fact that the psychoactive plants with psychotropic constituents that have been phytochemically identified are not cultivated but can be obtained from the wild by anyone makes it difficult to regulate their abuse.

Perhaps this is where social pharmacology, the new discipline in clinical pharmacology, can be useful in helping with ways to deal with any harm that result from abuse.

### Social pharmacology

The term “social pharmacology” was coined in 1960 as a methodology for describing addiction, effect on mood, and the behaviour of individuals in society. Over time, its methodology has improved, and it has now expanded to the knowledge and appreciation of how psychotropic drugs are accessed, used, and abused in the social life cycle and how to monitor their impact on public health (Morgan, 2016). Social pharmacology can also be applied to ethno-psychoactive plants such as those identified in the present study.

There will clearly be changes in the demography of the subgroups who will use these indigenous psychoactive plants*.* A booming industry, such as with cannabis, will certainly evolve to meet the demands of various types of consumer, as will the development of new products derived from ethno-psychoactive plants. Furthermore, these products will also have unknown health effects. Therefore, laws and attitudes need to change; data will be required as evidence for both the therapeutic and adverse effects of these ethno-psychoactive plants. Such data should be used as evidence in the prevention of hazardous use and in the maximization of potential medical benefits.

Given the historical controversies on *Cannabis*-based medicines and psychedelic plants and substances, rigorous attention is necessary to identify the psychotropic components of these African species and to investigate whether biphasic dose-response or other idiosyncratic properties are operative. Given possible damage to the cognitive functions of the brain, their primary psychoactive and intoxicating components are not known. Some adverse effects have been seen with THC in *Cannabis* (Copper and Adinoff, 2019) which could also be the case with the constituents of ethno-psychoactive plants. Observation of how these adverse effects of THC on cognitive function of the brain may translate to humans underline the importance of considering patterns of usage that may be motivated by abuse. This appreciation will guide hypothesis formulation related to the neuro-cognitive impact of exposure.

**FIGURE 1:**
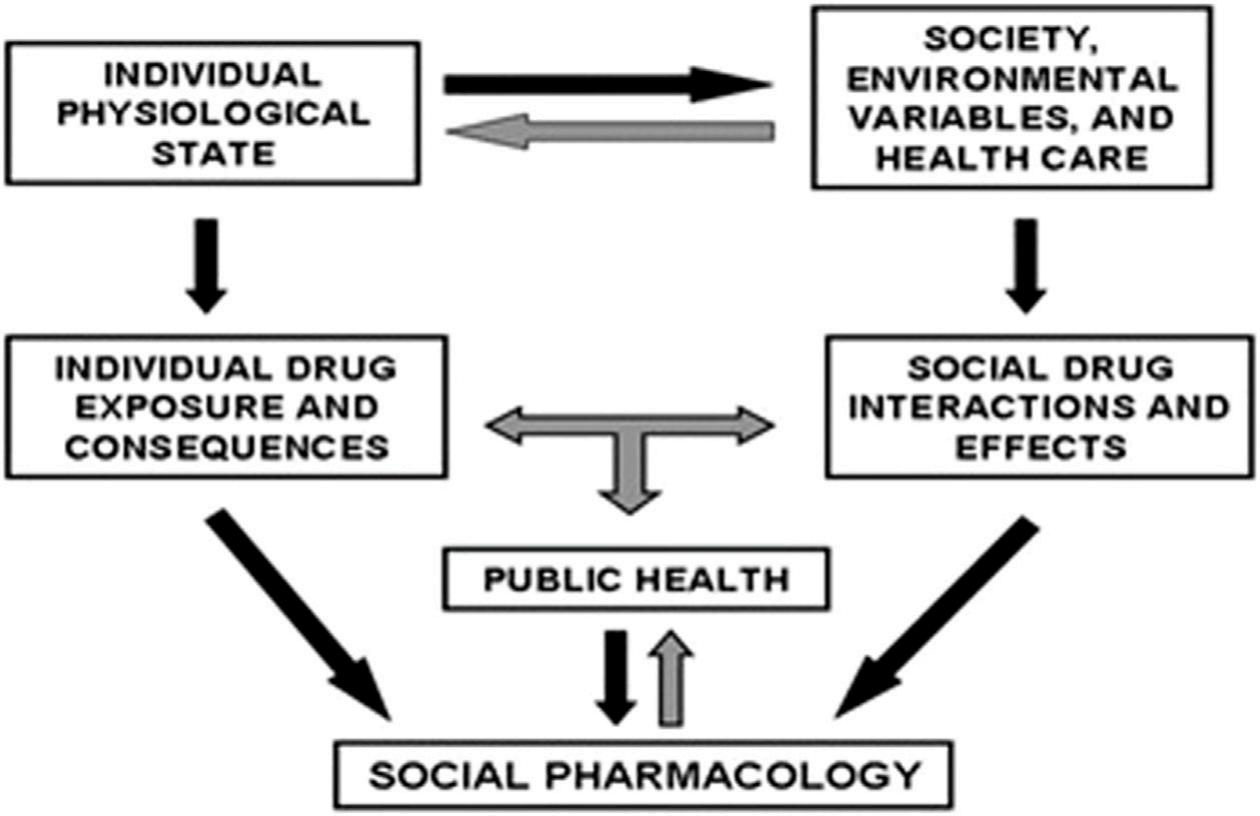
Inter-relationships important for social pharmacological methodology (Morgan, 2016).

There are, however, lessons that can be learnt from *Nicotiana tabacum*, currently from *Cannabis*, and perhaps from *Sceletium tortuosum.* There is also a need to translate our understanding of the positive and negative consequences of ethno-psychoactive plants. This is important from a policy perspective, especially when the pros and cons of implementing regulatory measures are clearly understood. Figure 1 summarizes the interrelationship between society, the public, and ethno-pharmacology that needs to be understood.

It cannot be over emphasized that an understanding of neurocognitive effects, whether positive or negative, is critical in the context of medical use to optimize any products that are developed. This is also important when it comes to clinical utility and mitigating undesirable outcomes such as intoxication and abuse.

During the interviews with TMPs, interesting terminology common to most Bantu vernacular languages meaning “to see”, was used to describe the effects of psychoactive plants. The plant would then be described as a plant that makes “one see,” analogous with metaphysical “seeing,” transcendental enlightenment, revelation, and the ability to arouse ancestral spirits. The veil of secrecy about these plants has now been lifted, and the general community is aware of these plants with such properties; hence, the pervasive desire to alter their consciousness.

### The most commonly used plants

The following psychoactive plants were found to be commonly used and had the potential for abuse.

#### *Boophone disticha* (L.f) Herb*, Amaryllis disticha* Amaryllidaceae

**FIGURE 2:**
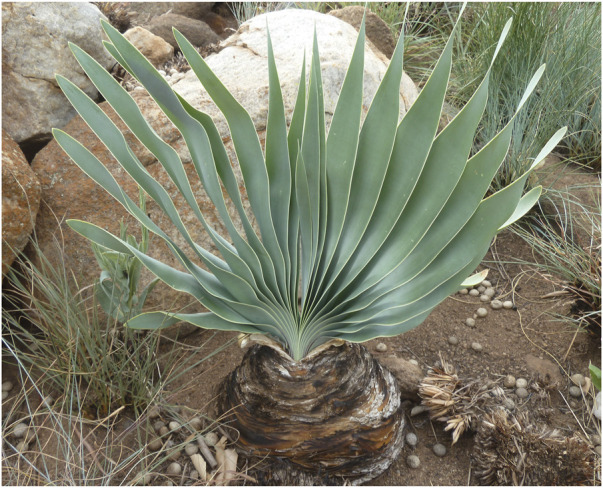
Leafy *B. disticha* showing a scaly exposed bulb.

*B. disticha* (Figure 2) is regarded as a visionary plant called *leshoma* in Sesotho (Pasquali, 2021). It is used as a divinatory “bioscope”, giving the ability to see things that ordinary people cannot see during consultation, by Zulu, Xhosa, Shona, and San TMPs. Each ethic group has a particular name for it.

During consultation with a traditional healer, a preparation of *B. disticha* is administered to the enquirer. This causes a visionary cataleptic state during which they can “see” the person responsible for their misfortune”. Over the years, such activities have been kept secret by TMPs, but now it is common knowledge in some communities. As a result, careless consumption by adolescents of a *B. disticha* decoction made from the bulb, both for therapeutic and intoxicating purposes—a herbal high—is now reported to be common in some communities (Laing, 1979).

The route of administration is as a liquid preparation of the bulb, usually administered orally.

The decoction from the bulb must be carefully prepared by boiling it at least thrice and discarding the boiled water containing toxic constituents. The last boil is then left to stand over night before being used (Nyazema, 1984).

To date, the presence of 11 alkaloids—including buphanidrine 13 (19.4%), undulatine 14 (18.6%), buphanisine 15 (16.9%), buphanamine 11 (14.1%), nerbowdine 12 (11.1%), crinine 3 (7.2%), distichamine 16 (5.4%), crinamidine 17 (1.2%), acetylnerbowdine 18 (0.6%), lycorine 2 (0.4%), and buphacetine (0.3%)—has been identified and described (Nair and Staden, 2014) with their respective contribution percentage. There is uncertainty over which of the 11 alkaloids is affected by the boiling of the bulb, which thereby influences the “entourage effect” described by observed symptoms including unconsciousness, dilated pupils, tachycardia, raised blood pressure, slightly raised temperature, laboured respiration, psychosis, drunkenness, and visual disturbances (Laing, 1979).

Stafford et al. (2008) reported that *B*. *disticha* can be used for phytotherapy for mental disorders such as anxiety, depression, epilepsy, and age-related dementia.

**FIGURE 3:**
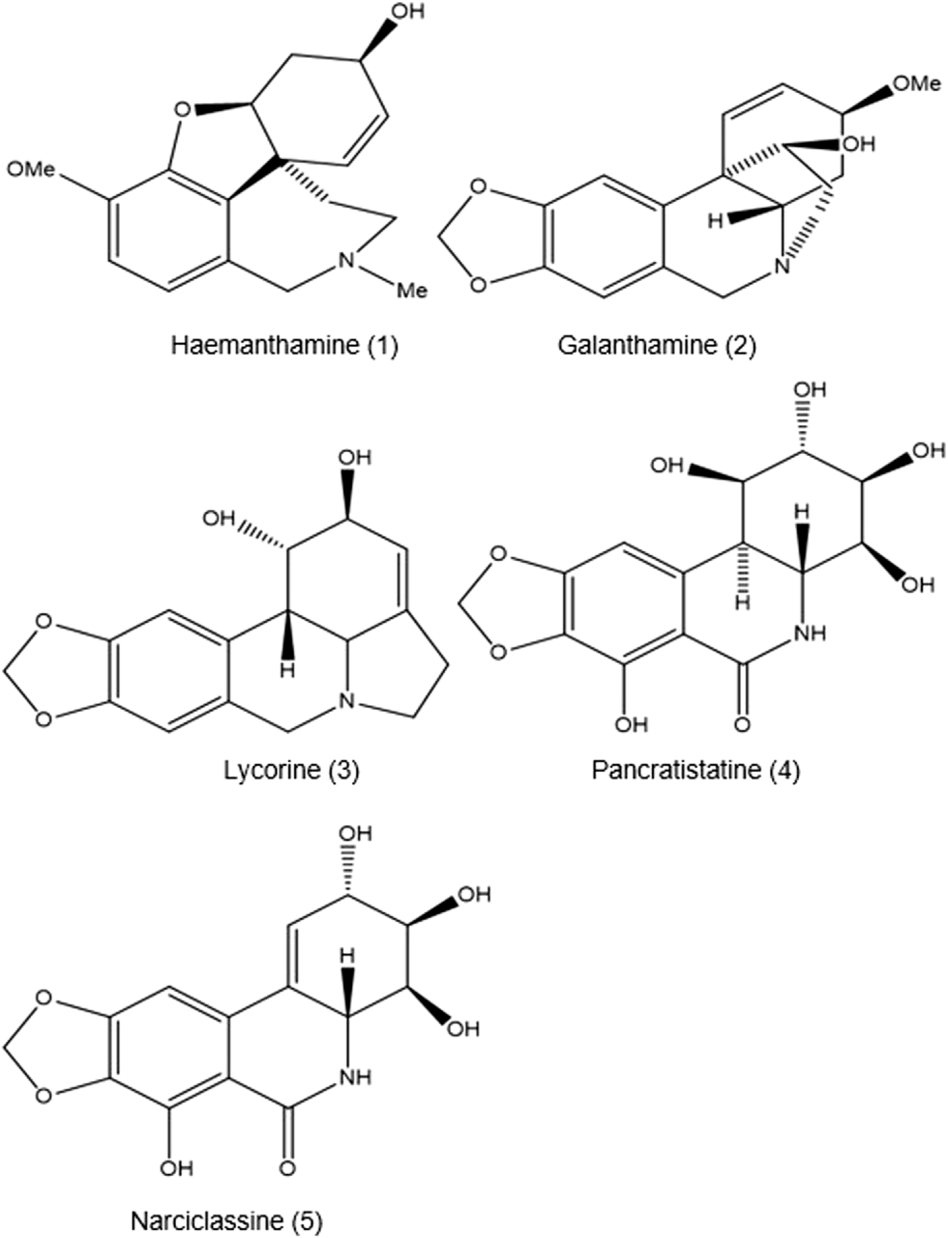
Amaryllidaceae alkaloids.

All the alkaloids mentioned above produced by *B. disticha* are of the crinane series. As shown in Figure 3, nerbowdine, previously called “haemathamine” (Gelfand and Mitchell, 1952), was thought to be responsible for effects similar to those of anti-cholinergic scopolamine.

Several areas of *B. disticha* use in traditional medical practice still require pharmacological validation because the plant is regarded as poisonous. There is a need for rigorous investigation to reduce harm that may be caused by the use and abuse of *B. disticha* (Stafford et al., 2008).

There are no legal sanctions in southern African countries, which opens the drug to possible abuse.

#### *Cannabis sativa*, L Cannabaceae

**FIGURE 4:**
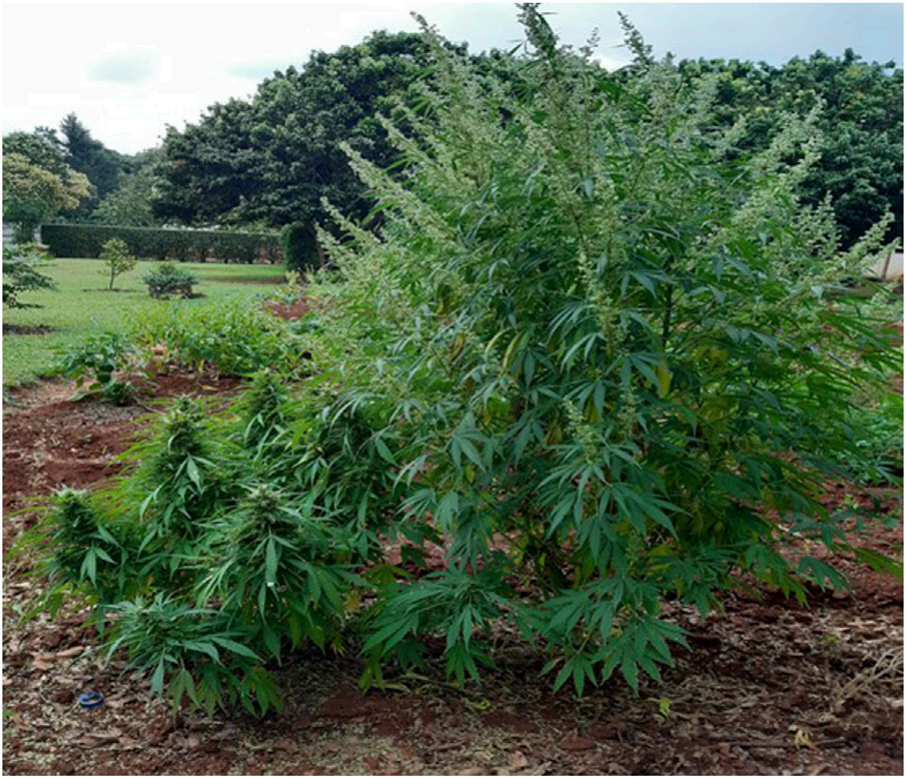
Backyard female (l) and male (r) *Cannabis*.

*C. sativa* is not indigenous to southern Africa, as believed by many people. A more in-depth analysis of the issue of cannabis diffusion in Africa versus its possible status as a native plant has been extensively reviewed in a brief agricultural history of Africa by Duvall (2019). Linguistic evidence indicates that it was introduced into the sub-continent from Asia (Crampton, 2015; du Toit, 1996). Be that as it may, *Cannabis* is now reported to be used by many TMPs (Sobiecki, 2008). Illegal use by non-healers for recreation and ritual is widespread throughout southern Africa. However, South Africa is moving towards the decriminalization of its cultivation for possession personal use. Figure 4 shows a female and a male plant in a household backyard, which is now permitted.

The major route of administration is smoking as a cigarette. It is believed that cannabis may be mixed with *N. tabascum* and other plants to aid divination and its psychotropic effects. Vaporization is another commonly used route of administration. Inhalation is believed to be quickest for onset of action and shortest duration (Iversen, 2001).

**FIGURE 5:**
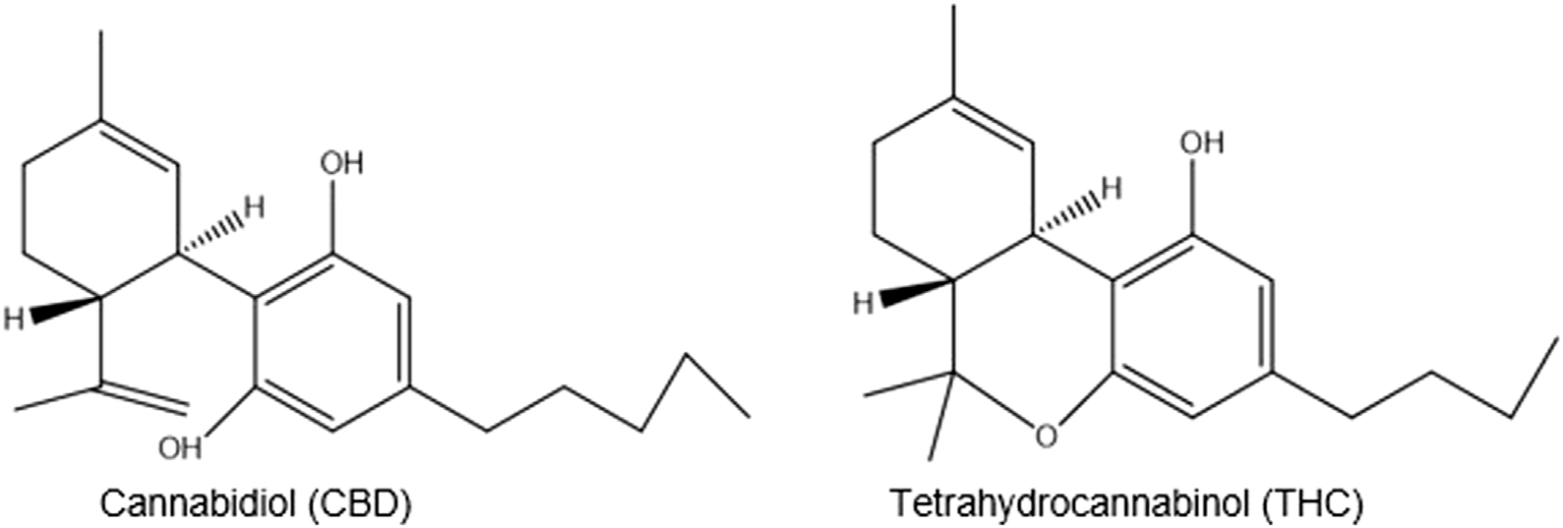
Chemical structures of CBD and THC.

*C. sativa* is used for religious purposes as a sacred herb entheogen by southern African Rastafarians who call it *ganja (ganga)*, a word derived from Indian plantation workers in Jamaica (Booth, 2005; Crampton, 2015). It is mainly used for its THC (tetrahydrocannabinol) content, which is responsible for its intoxicating effects. More than 100 other cannabinoids including, CBD (cannabidiol), are non-intoxicating (Figure 5) (Russo, 2017).

Cannabis grown in different conditions can produce different phytochemical profiles which may not be known by its users. Studies have shown that handling of the plant can cause the oxidation and degradation of cannabinoids, resulting in adverse or unknown biological effects. This is critical information of primary importance for its medical and recreational use (Zandkarimi, Decatur, Casali, Gordon, Skibola, et al., 2023).

There are cannabinoid-like substances in the brain that can interfere with or modulate neurotransmission. This is because cannabinoid-like substances bind to CB1 receptors on the pre-synaptic neuron found in the brain. Depending on the neurotransmitter released as a result of the binding, this can result in different psychotropic effects, depending on the part of the central nervous system (Mouhamed et al., 2018).

There is continued research into the psychological effects of cannabis . An interesting recent study has shown that the volume of the grey matter of the brain increased following a couple of uses of marijuana by some adolescents, resulting in increased risk of anxiety, and decreased ability to think and memory (Orr, Spechler, Cao, Albaugh, Chaarani et al., 2019). The genetic basis for cannabis use and its psychiatric disorders has been published in *Lancet Psychiatry*, indicating that a subset of the population is at high risk (Cheng, Parker, Karadag Koch, Hindley et al., 2023).

### Its pharmacological classification is as a stimulant and sedative

The legal status of *C. sativa* in southern Africa is that it is generally illegal for adult (recreational) use in most countries, but there is a decriminalization drive.

#### *Datura stramonium,* L Solanaceae

The early Sanskrit word *dustura* or *dahatura* means “divine” inebriation, from which *D. stramonium* derives its name. This explains why, for approximately 400 years, solanaceous plants were regarded as “diabolic incarnations”. Consequently, *Datura* has been referred to as “devil’s apple”, “mad apple”, and “devils work” due to its ability to cause visionary dreams and assist in the foretelling of the future and revealing the causes of disease (Busia and Heckles, 2006).

**FIGURE 6:**
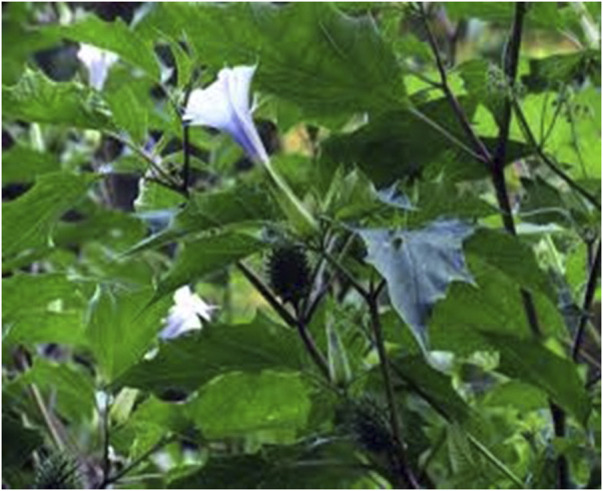
*Datura* with flower-like trumpet.

In southern Africa *D. stramonium* has been reported to be used in traditional medical practice (Figure 6) (Sobiecki, 2008). It is thought that the use of its leaves can relieve headache, and vapours from a leaf infusion can relieve the pain of rheumatism and gout. The smoke of the leaf can be inhaled to relieve asthma and bronchitis.

### Route of administration: inhalation

Hysterical and psychotic patients are sedated by using its seeds and leaves. *D. stramonium* is also thought to help in insomnia. The seeds are reported to be its most medicinally active part (Soni, Siddiqui, Dwivedi and Soni, 2012).

Conscious perception only occurs when the associative cortex of the brain is active. Different structures of the brain have been reported to be responsible for different levels of consciousness. Different neurotransmitters are involved in these, which means that interference with these neurotransmitters at the synapses will affect the consciousness of an individual. The cholinergic system, whose neurotransmitter is acetylcholine, is also responsible for major neuromodulatory systems involved in relaying information between brain structures (Ach) (Roth, 2004).

**FIGURE 7:**
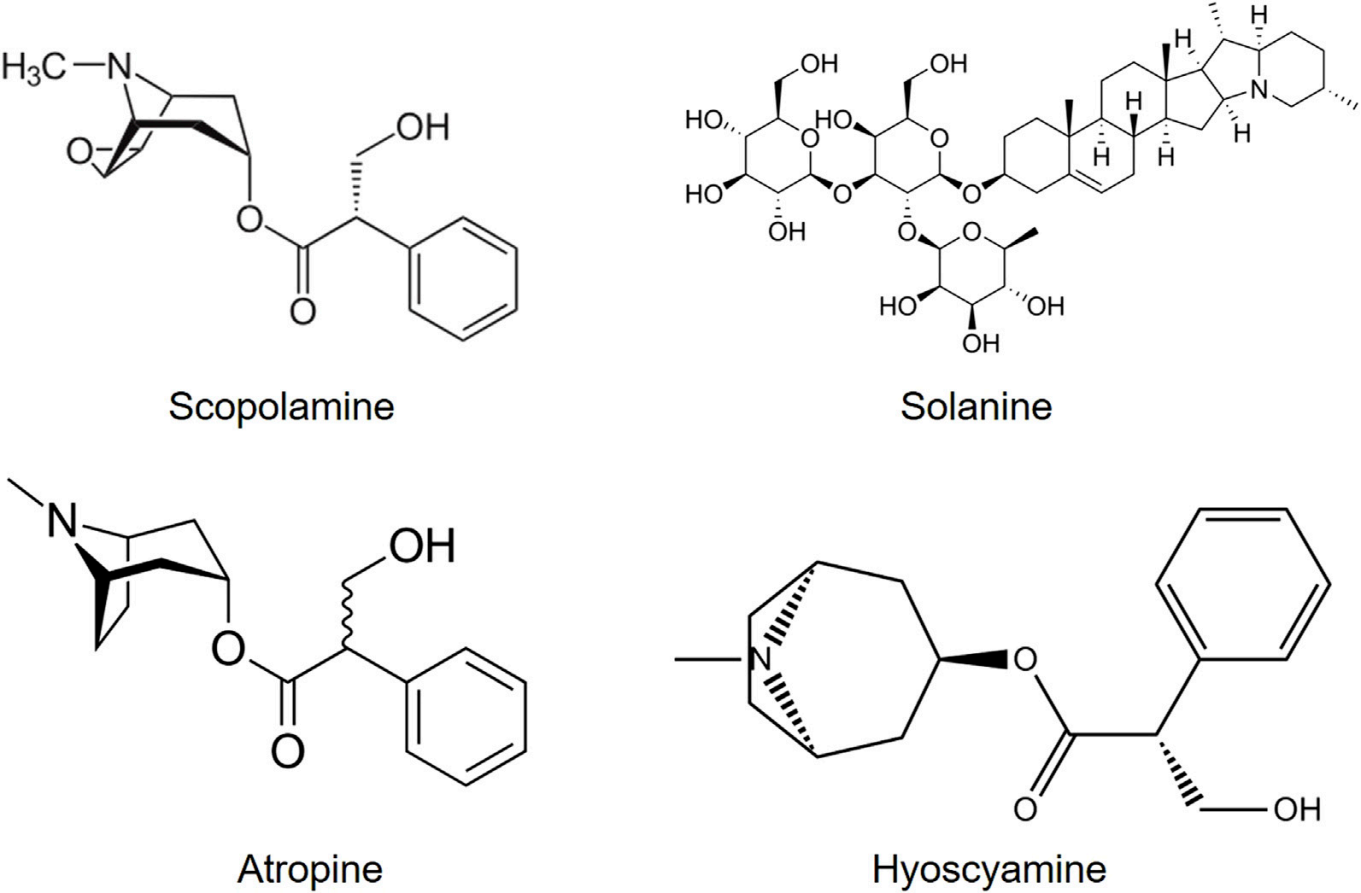
*Datura* alkaloids.

All parts of *Datura* are reported to contain dangerous levels of tropane alkaloids, atropine, hyoscynamine, and hyoscine (scopolamine) (Figure 7) which can stimulate no-cost highs. The effects of *D. stramonium* have been postulated to derive from the major alkaloid responsible for the principal effects of hyoscine. The tropane alkaloids work additively to trigger hallucination (Hall, Pfefferbaum, Gardner, Stickney and Perl, 1978).

legal status: no legal restrictions despite potential harmful and deadly effects.

#### *Helichrysum odoratissimum* L. asteraceae sweet. african incense, aromatic shrub

**FIGURE 8:**
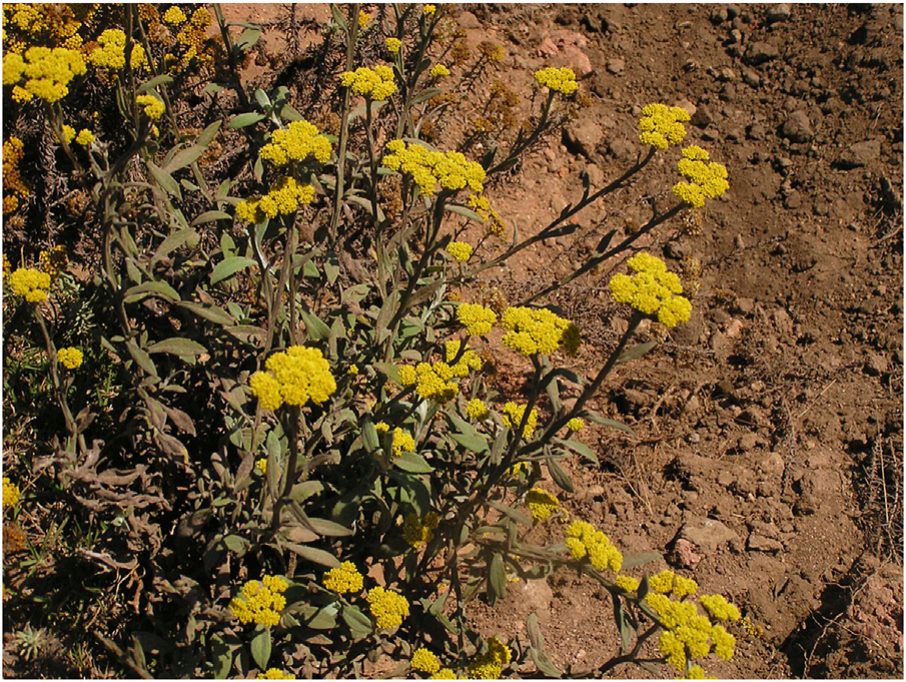
*H. odoratissimum* in the wild.

The name of *H. odoratissimum* is derived from the Greek word *heliscryson*, a compound of the word for “sun” plus *chryos*, which means “gold,” referring to the golden flowers which are characteristic of the genus (Figure 8). “*Odoratissimum*” refers to the strong odour of the plant. The plant is commonly termed “African incense”, and perhaps this is why it is used as a calmative for insomnia and ritual incense (Maroyi, 2019; Serabele, Chen and Combrinck, 2023). Recently, it has been reported that another species in the *Helichrysum* genus, *H. umbraculigerum*, which is not commonly used, produces phytocannabinoids such as cannabigerol and CBG but not THC and CBD (Benson, 2023). TMPs in southern Africa have their own ways of distinguishing the different *Helichrysum* species, in spite of their florescence being almost identical (Figure 8) (Viljoen et al., 2022).

The principal route of administration is the inhalation of smoke from burnt parts of the plant or direct smoking. It can, however, also be taken as herbal tea.

**FIGURE 9:**
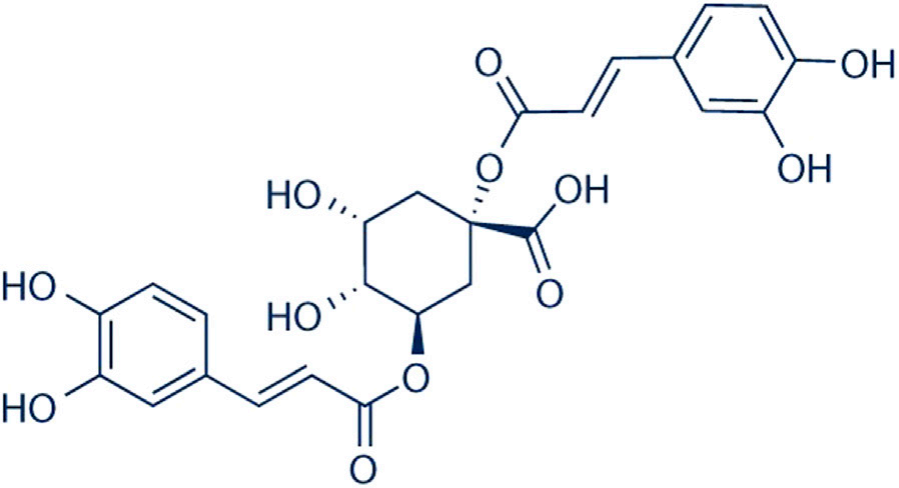
Dicaffeoylquinic acid.

According to the phytochemical studies of Viljoen et al. (2022), 4,5-dicaffeoylquinic acid and dicaffeoylquinic acid, also found in coffee beans (Mondolot et al., 2006) (Figure 9), consistently appear to be possible markers for the species. This has also been confirmed by Serabele, Chen and Combrinck (2023), who used high-performance-thin-layer-chromatograph (HPTLC) coupled with mass spectrometry (UPLC-MS) analysis. What is interesting, however, is that essential oils such as α-pinene-containing oils and neryl acetate also may be possible markers of the species, using the method of analysis of Tundis, Statti, Conforti, Bianchi, Agrimonti et al. (2005). Their study of *Helichrysum italicum* showed that environmental factors influenced the phytochemistry (volatile constituents) and biological effects (antibacterial activity) of the plant. Interestingly, *H. italicum* is commonly used as a herbal medicine in Mediterranean regions (Appendino et al., 2015).

There are no legal restrictions on its use.

#### *Leonotis leonurus* L. R. Br Lamiaceae

*L. leonurus,* although often referred to as “wild dagga,” an Afrikaans word for cannabis, is not related to true dagga (*C. sativa*). *Leonotis* is a Greek word meaning “lion ear”.

**FIGURE 10:**
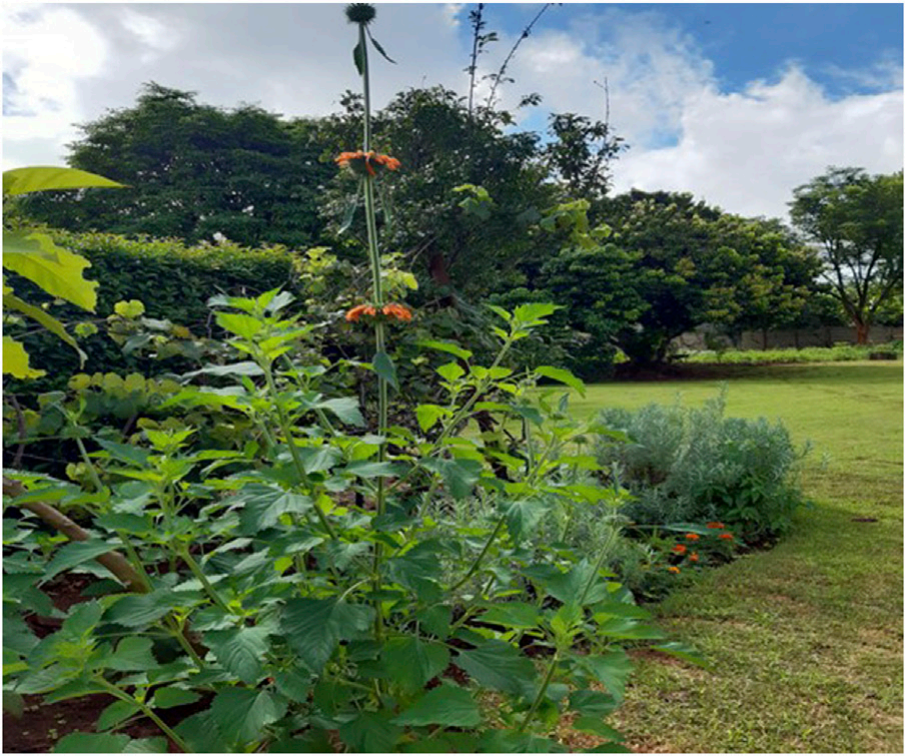
*L. leonurus*.

In traditional medical practice, its dried leaves and pink or brilliant orange flowers, (Figure 10) are used to treat many ailments, including epilepsy. Non-healers use it for its calming effect.

Route of administration: The leaves and flowers are smoked for mild euphoric effect less potent than cannabis.

**FIGURE 11:**
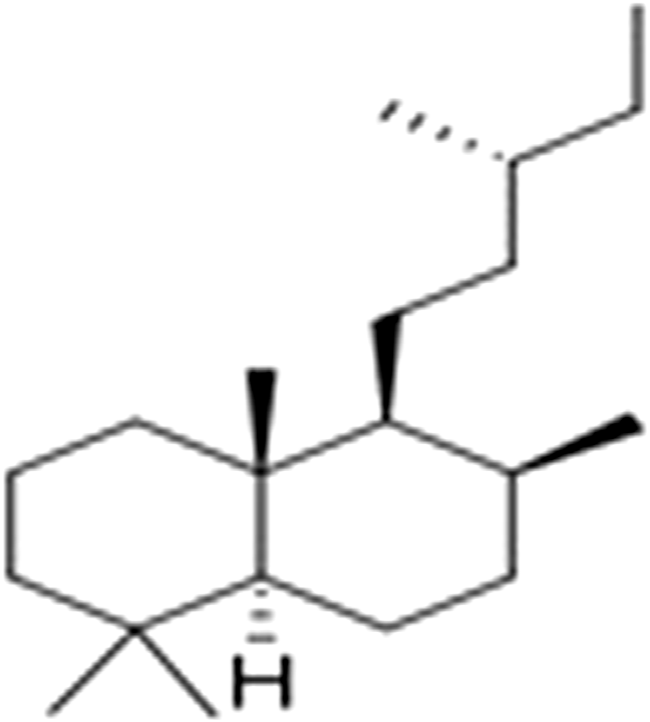
Labdane.

Terpenoids with the main compounds (mono-, sesqui-, and diterpenoids) have been reported to be biologically active. Labdane diterpenes (Figure 11) were found to be more abundant compounds by Nsuala, Enslin and Viljeon (2015).

**FIGURE 12:**
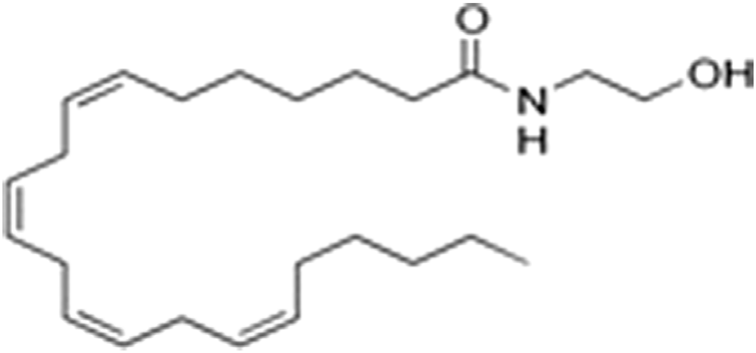
Adronylethanolamine (Adrenyl-EA).

**FIGURE 13:**
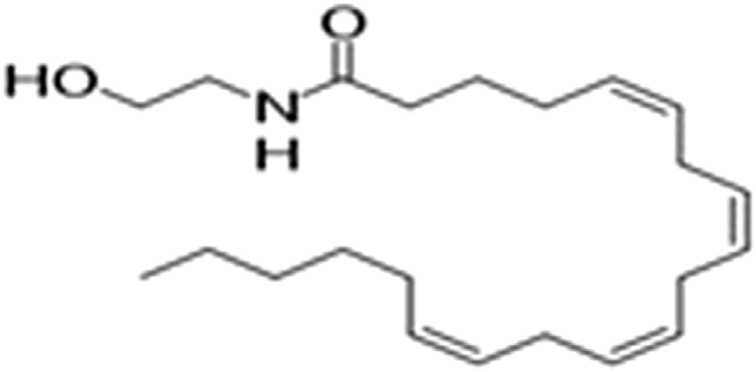
Arachidonylethanolamine (anandamide).

However, a phytocannabinoid-like compound, adrenoyl-EA (adronyl-ethanolamine), (Figure 12) has recently been identified in the flowers. The compound has the same structure as endocannabinoid anandamide (N-acylethanolamine) (Figure 13) which is also found in cocoa beans. Anandamide is as an agonist of the CB1 and TRPV1 receptor proteins in humans, whose other agonists are ideal candidates for the development of anti-inflammatory, neuroprotective, and anticancer drugs (Hunter, Stander, Kossman, Chakraborty, Prince et al., 2020).

**FIGURE 14:**
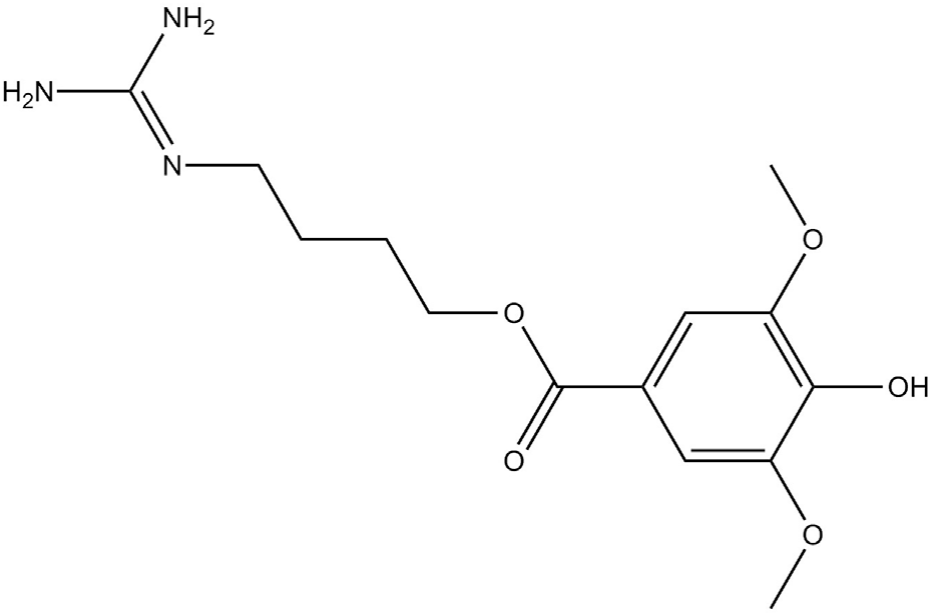
Leonurine.

There are other compounds that have been isolated from the plant. However, according to Nsuala, Enslin and Viljeon (2015), leonurine (Figure 14) has been reported in the popular literature. Interestingly there has been no scientific analysis of extracts of *L. leonurus* that have reported leonurine.

Legal status: no restrictions on its use.

#### *Psilocybe cubensis* (Erale) Singer. Hymenogastraceae

**FIGURE 15:**
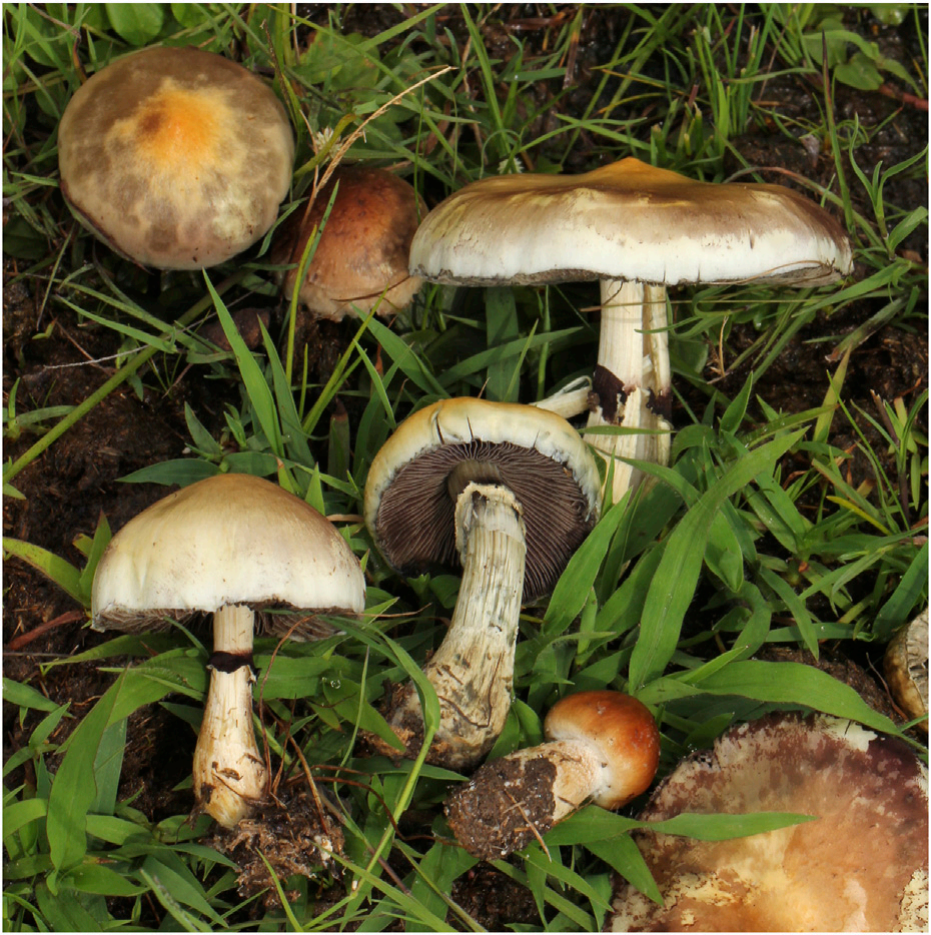
*P. cubensis*.

*P. cubensis* is a fungus with neurotropic (hallucinogenic or psychotropic) properties, also referred to as narcotic, magic, sacred, psychedelic entheogenic mushrooms; they have been reported as found all over the world, including southern Africa (Matsushimaet al., 2009). Because of the legal status of mushrooms use, coupled with continued secrecy surrounding some traditional medical practices, it was difficult to obtain information on how *P. cubensis* (Figure 15) is actually employed in culturally sanctioned visionary experiences in ritual or religious contexts.

Its route of administration is oral.

This is also the case in West Africa (Osemwegjie, Okhuoya and Dania, 2014). Ethnomycological studies in some parts of Africa have been found to be varied, incoherent, skewed, and not balanced in terms of information on mushrooms of unknown use.

However, information obtained about the collection and uses of mushrooms indicate that there is community knowledge about which mushrooms can be eaten, which can be used for medicinal purposes, and which are poisonous. There are beliefs such as the following: 1) any wild mushroom attacked by insects, rodents, and other animals where they are growing can be classified as edible; 2) any mushroom given to a live chicken, pig, dog, and which does not die is regarded as edible; 3) brightly coloured and pleasantly smelling mushrooms could be edible and associated with spirituality. Such human experiments provide more knowledge about the better use of wild mushrooms in general, leading to their acceptance as food, medicine, or poison.

*P. cubensis*, which is reported to grow on cow and wildlife dung in Zimbabwe (van der Horst, 2018), is similar to that which grows on Asian elephant dung in India. Psilocybin, psilocin (Supplementary Figure S1), and baeocystin are reported to be the biologically active substances in the mushroom. Of these biologically active substances, psilocybin is the most stable. It is found in some of the mushrooms and is converted to psilocin, which produces the psychoactive effect (Stamets, 1996). The activity produced by the mushrooms depends on the species and variety and where it grows and is subsequently harvested for use. The same is true with any of the psychoactive plants. There are currently several clinical trials with psilocybin (University of San Francisco, 2023). Previous studies at the Johns Hopkins Centre for Psychedelic and Consciousness Research showed that psilocybin could be effective for therapeutic purposes, such as for depression, drug abuse, and end-of-life mental disorders (Ziff et al., 2022). Despite this, “magic mushrooms” are easily accessible to the general public.

#### *Mesembryanthemum tortuosum, Sceletium tortuosum*. L. N.E.brown Aizoaceae (kanna) mesembryanthemaceae

*S. tortuosum* L.) N.E. Br. and *S. expansum* L. Bolus were formerly known as *Mesembryanthemum tortuosum* L. and *Mesembryanthemum expansum* L. (Supplementary Figure S3). *Sceletus* is a Latin word that describes the plant’s prominent leaf veins (Supplementary Figure S4).

A number of habitats have been found suitable for the growth of different genera of its family. It is found in south-west Africa, including Angola, South Africa, Zimbabwe, Botswana, and Namibia (Supplementary Figure S4) (Faber, Laubscher and Jimoh, 2021).

Traditionally, *S. tortuosum* was reported to be useful for toothache and abdominal pain. Furthermore, it is thought to elevate mood, suppress hunger and thirst, induce analgesia, aid hypnosis, reduce anxiety, is used as an intoxicating/euphoric substance (Olatunji et al., 2021), and is sometimes used together with cannabis to enhance the latter's intoxicating effect (Michell, 2004).

The route of administration is mainly oral by mastication, and by inhalation through smoking the dried plant material or snorting the root powder.

The plant contains different types of mesembrine alkaloids (Supplementary Figure S5) which are found in a few plant genera, *Sceletium* being one (Faber, Laubscher and Jimoh, 2020).

Mesembrine is a serotonin re-uptake inhibitor and is also thought to act as a monoamine-releasing agent (Coetzee et al., 2016). Historical use of different extracts of the plant by the San and Khoi people has been shown to have various biological properties (Manganyi et al., 2021).

It can therefore be argued that *S. tortuosum* has both pharmaceutical and economic significance because it contains mesembrenone and mesembrine, which can be developed as useful products that promote health and/or to treat some psychological disorders.

#### *Silene capensis* Caryophyllacae, African Dream root

According to anthropologist Manton Hirst, *S. capensis* has been used for hundreds of years. It is a stringy, leafy green plant found near rivers that produces a fragrant white flower that only blooms at night, hence perhaps its name as the “African dream herb” (Hirst, 2005). It is believed that *S. capensis* is used to heighten one’s intuitive capacity to extract wisdom from the dream realm and apply it the waking state. Details of how *S. capensis* is used depend on the Bantu ethnic group, including how the plant material preparation is consumed.

The plant’s route of administration is oral, by chewing the root bark, or scooping or sipping the foam that it produces during preparation.

*S. capensis* is used in ritual ceremonies; hence, the potential for abuse because information on accessibility and availability is now public knowledge. Perceptions regarding its value and potential harm have been reported to be transformed by those participating in ceremonies.

Most *Silene* species are hermaphrodite, and more than 400 bioactive compounds have been isolated, including phytoecdysteroids, the predominant constituents (Mamadalieva, 2012). Supplementary Figure S6 shows the general chemical structure of phytoecdysteroids, and Supplementary Figure S7 gives the structure of 20-hydroxyecdysone.

Ecdysteroids can be used for chemotaxonomy in this genus because many *Silene* species contain complex ecdysteroid cocktails (Zibareva et al., 2009). Despite what is now known about the responsibility of 20-hydroxyecdysone for genomic biological effects manifested in different animal models (Dinan, Dioh, Veillet, and Lafont, 2021), ecdysteroids are thought not to be responsible for CNS effects.

According to Sparg, Light, & van Staden, (2004), the frothing and foam formation associated with preparation for use could be explained by the presence of saponins— oleanane-type aglycone moieties. Supplementary Figure S8 shows the basic structure of a saponin said to be commonly present in the Caryophyllacae order. Since the foam is generally consumed during the ceremonies, it is therefore assumed that its psychoactive effects are due to the saponin content rather than the phytoecdysteroids.

Observations made by Hostettmann & Marston (1995) confirm that cleavage of the saponins aglycone moiety can serve as a useful substance for the synthesis of hormones such as progesterone in industry. The amphiphilic nature of saponins is useful in soap manufacture and surfactants for the cosmetic industry (Guclu-Ustundag and Mazza, 2007).

Ground root is now commercially available in gel capsules which contain approximately 500 mg of ground powder of the root. It is promoted for lucid dreams as well as enhancing the quality of sleep, with a maximum daily dose of 30 g (Sumpter, 2021).

There are no legal restrictions on its use

#### *Dioscorea dregeana* (Kunth) T. Durand & Schinz

*D. dregeana* (wild yam) (Supplementary Figure S9) grows naturally in Eswatini, Mozambique, and South Africa. It is mainly used as a sedative, depending on the mental disorder (Maroyi, 2022).

Its route of administration is oral as a weak decoction which is a more vigorous extraction of the active ingredient from the plant’s tuber.

The tubers of *D. dregegeana* contain the following compounds: sitosterol, stigmasterol, dodecanosyl 3-[4′-hydroxy, 3′-methoxyphenyl] propenoate, 3, 4′,5-trihydroxybibenzyl, crinamine, and dioscorine. Of these phytochemicals, dioscorine (Supplementary Figure S10) has intoxicative and soporific effects. On the other hand, crinamine (Supplementary Figure S11) is a selective monoamine B inhibitor (Naidoo et al., 2020).

An animal study found that a combination of the psychoactive principles in the tuber produced central nervous system depressant/sedative and anxiolytic effects (Patel and Galani, 2017). Bio-prospecting of *D. dregeana* and wild tubers in the food and pharmaceutical industries can benefit from the observed effects.

There are no legal restrictions on its use.

#### Potential pharmaceutical development with ethno-psychoactive plants

Over the years, the commercialization of tradition African medicinal plants has increased as a result of interest in and knowledge of their use. For example, the economic value of indigenous medicinal plants in South Africa now represents 5.69% of the national health budget, and the plant trade is a key rural industry and business stimulant (Rispel and Setswe, 2007). It is therefore necessary to investigate ethno-psychoactive plant use for their psychotropic actions.

These plants contain secondary metabolites including terpenoids, flavonoids, and alkaloids. These metabolites feature various chemical structures and produce a variety of beneficial biological which are a valuable source of compounds that the pharmaceutical, nutraceutical, cosmetic, and fine chemical industries can use. The present study shows that ethno-psychoactive plants are a major source of many drugs that can be developed for therapeutic purposes. Many of these plants contain useful substances, either in the whole plant or in some parts that can used be for therapeutic purposes in disease, diagnosis, and prevention. It has been estimated that 84% of drugs or their structures now used for mental disorders have been obtained from natural sources (Bharate et al., 2018). According to Halilu (2022), medicinal plants can be a significant source of income for research in pharmaceutical science and socio-economic development. *B. disticha* is certainly a plant from which neuro-protective products can be developed for Alzheimer’s and Parkinson’s diseases, epilepsy, depression, and anxiety. The crinamine contained in *B. disticha* has been demonstrated to be a selective inhibitor of monoamine oxidase B, which is useful in the treatment of Parkinson’s disease. Another plant which contains crinamine, *Crossyne guttata,* is used by Rastafarian bush doctors for alcoholism, which is accentuated by MAO (Naidoo et al., 2020).

Research on treatments for depression, post-traumatic stress disorder, and other psychiatric conditions using psychedelics has been brought closer to legalization (Krediet et al., 2020).

Another group of psychoactive plants, the *Silene* genus, has been exploited as an industrial source of phytoecdysteroids that can be used to develop anabolic steroids. *S. capensis*, the dream root, is another source of hallucinogens that can be developed for specific mental disorders and to improve the quality of sleep. *C. sativa* (THC), *D. stramonium, N. tabacum*, and *P. cubensis* can be exploited just as *C. sinensis* and *N. tabascum* have been by industry. The recent discovery that non-cannabis plants such as *H. umbraculigerum* have cannabis qualities opens new avenues for the development of products of medical use, particularly psychiatrics (Berman et al., 2023). This species has been shown to have a parallel evolution of cannabinoid biosynthesis which can be exploited to produce botanicals for medicinal use.

On the other end of the spectrum, psychosis—a collection of symptoms affecting the mind, where there has been some loss of contact with reality—can be treated and managed in traditional medical practice. Psychosis in traditional medical practice is regarded as a disequilibrium that results from psychological or spiritual factors or both. It is also referred to as a collection of symptoms that affect the brain, causing a loss of contact with reality. The healing of such patients emphasizes correcting this disequilibrium by using *C. sativa*, *D. dregeana, S. tortuosum, L. leonurus,* and *H. odoratissimum*. Unfortunately, little is being done to authenticate their effects within a scientific paradigm because they do not contain a simple ingredient, making them more complex than conventional pharmaceutical products like prochlormazine. The many substances that may be contained in a plant responsible for beneficial effects may act synergistically and produce what is known as “entourage effect” (Ferber, Namdar, Hen-Shoval, Eger, Hinanit Koltai, et al., 2020). This synergistic interaction may be a strength for medicinal plants, but unfortunately it is also an obstacle to standardizing research, which is a concern for those trying to develop pharmaceutical products from medicinal plants. It should be a concern for TMPs, conventional healthcare providers, and consumers. Such complexity requires psycho-pharmacological and social pharmacological information on the value of botanicals used for mental problems, diagnosed in traditional medical practice, in the assessment of a risk-effective balance.

## Conclusion

TMPs in southern Africa commonly use indigenous herbal medicines for divination and also for to treat and manage mental and other illnesses. Unfortunately, the results of research, risks, and benefits from their use do not align. Psychoactive plants could be explored further to develop therapeutic agents. Although the CNS effects of the psychoactive plants identified are known, little is known about their potential for abuse among TMPs and in some communities in southern Africa. There is therefore a need to work closely with TMPs to reduce harm from the abuse of these plants while also promoting pharmaceutical development. This continues to be an under-investigated area that deserves continued scientific enquiry.

## References

[B1] Appendino G. B. Pollastro F. Minassi A. Andrea M. Ballero M. (2015). Helichrysum italicum: sleeping giant of the Mediterranean herbal medicine. Herbalgram 105, 36–47.

[B68] Adinoff B. Cooper Z. D. (2019). Cannabis legalization: Progress in harm reduction approaches for substance use and misuse. The American Journal of Drug and Alcohol Abuse 45 (6), 707–712. 10.1080/00952990.2019.1680683 31755837

[B2] Benson P. (2023). Plant with cannabis qualities opens new avenues for medicinal use. Available at: https://www.jns.org/plant-with-cannabis-qualities-opens-new-avenues-for-medical-use/.

[B3] Berman P. de Haro L. A. Jozwiak A. Panda S. Pinkas Z. Dong Y. (2023). Parallel evolution of cannabinoid biosynthesis. Nat. plants 9, 817–831. 10.1038/s41477-023-01402-3 37127748

[B4] Bharate S. S. Mignani S. Vishwakarma R. A. (2018). Why are the majority of active compounds in the CNS domain natural products? A critical analysis. J. Med. Chem. 61 (23), 10345–10374. 10.1021/acs.jmedchem.7b01922 29989814

[B5] Booth M. (2005). Cannabis: a history. Macmillan.

[B6] Busia K. (2006). Jimson weed: history, perceptions, traditional use and potential therapeutic benefits of the genus datura. Herbalgram 69, 40–50.

[B7] Cheng W. Parker N. Karadag N. Koch E. Hindley G. Icick R. (2023). The relationship between cannabis use, schizophrenia, and bipolar disorder: a genetically informed study. Lancet Psychiatry 10 (6), 441–451. 10.1016/S2215-0366(23)00143-8 37208114 PMC10311008

[B8] Coetzee D. D. López V. Smith C. (2016). High-mesembrine Sceletium extract (Trimesemine™) is a monoamine releasing agent, rather than only a selective serotonin reuptake inhibitor. J. Ethnopharmacol. 177, 111–116. 10.1016/j.jep.2015.11.034 26615766

[B9] Crampton H. (2015). Dagga: a short history:(then, now & just now). Johannesburg, South Africa: Jacana.

[B10] Dall’Acqua S. (2013). Plant-derived acetylcholinesterase inhibitory alkaloids for the treatment of Alzheimer&amp;#39;s disease. Botanics Targets Ther., 19–28. 10.2147/btat.s17297

[B11] Dinan L. Dioh W. Veillet S. Lafont R. (2021). 20-Hydroxyecdysone, from plant extracts to clinical use: therapeutic potential for the treatment of neuromuscular, cardio-metabolic and respiratory diseases. Biomedicines 9 (5), 492. 10.3390/biomedicines9050492 33947076 PMC8146789

[B12] du Toit B. M. (1996). Pot by any other name is still A study of the diffusion of cannabis. South Afr. J. Ethnology 19 (4), 127–135.

[B13] Duvall C. S. (2019). A brief agricultural history of cannabis in Africa, from prehistory to canna-colony. EchoGéo 48. 10.4000/echogeo.17599

[B14] Faber R. J. Laubscher C. P. Jimoh M. O. (2021). “The importance of sceletium tortuosum (L) NE brown and its viability as a traditional african medicinal plant,” in Natural drugs from plants (London, UK: IntechOpen).

[B15] Ferber S. G. Namdar D. Hen-Shoval D. Eger G. Koltai H. Shoval G. (2020). The “entourage effect”: terpenes coupled with cannabinoids for the treatment of mood disorders and anxiety disorders. Curr. Neuropharmacol. 18 (2), 87–96. 10.2174/1570159X17666190903103923 31481004 PMC7324885

[B16] Gelfand M. * Mitchell C. (1952). Buphanine poisoning in man. South Afr. Med. J. 26 (28), 573–574. 12984189

[B17] Güçlü-Üstündağ Ö. Mazza G. (2007). Saponins: properties, applications and processing. Crit. Rev. food Sci. Nutr. 47 (3), 231–258. 10.1080/10408390600698197 17453922

[B18] Halilu E. M. (2022). “Cultivation and conservation of african medicinal plants for pharmaceutical research and socio-economic development,” in Medicinal plants (London, UK: IntechOpen).

[B19] Hall R. C. Pfefferbaum B. Gardner E. R. Stickney S. K. Perl M. (1978). Intoxication with angel's trumpet: anticholinergic delirium and hallucinosis. J. Psychedelic Drugs 10 (3), 251–253. 10.1080/02791072.1978.10471882

[B20] Hewson M. G. (1998). Traditional healers in southern Africa. Ann. Intern. Med. 128, 1029–1034. 12_Part_1. 10.7326/0003-4819-128-12_part_1-199806150-00014 9625666

[B21] Hirst M. (2005). Dreams and medicines: the perspective of Xhosa diviners and novices in the Eastern Cape, South Africa. Indo-Pacific J. Phenomenology 5 (2), 1–22. 10.1080/20797222.2005.11433901

[B22] Hostettmann K. Marston A. (1995). Saponins. Cambridge, UK: Cambridge University Press.

[B23] Hunter E. Stander M. Kossmann J. Chakraborty S. Prince S. Peters S. (2020). Toward the identification of a phytocannabinoid-like compound in the flowers of a South African medicinal plant (Leonotis leonurus). BMC Res. Notes 13 (1), 522–526. 10.1186/s13104-020-05372-z 33172494 PMC7653773

[B24] Iversen L. L. (2001). The science of marijuana. Oxford University Press.

[B67] Jean-Francois S. (2014). Psychoactive plants: A neglected area of ethnobotanical research in Southern Africa. Studies on Ethno-Medicine 8 (2), pp.165–172. 10.1080/09735070.2014.11917631

[B25] Kohelová E. Peřinová R. Maafi N. Korábečný J. Hulcová D. Maříková J. (2019). Derivatives of the β-crinane Amaryllidaceae alkaloid haemanthamine as multi-target directed ligands for Alzheimer’s disease. Molecules 24 (7), 1307. 10.3390/molecules24071307 30987121 PMC6480460

[B26] Krediet E. Bostoen T. Breeksema J. van Schagen A. Passie T. Vermetten E. (2020). Reviewing the potential of psychedelics for the treatment of PTSD. Int. J. Neuropsychopharmacol. 23 (6), 385–400. 10.1093/ijnp/pyaa018 32170326 PMC7311646

[B27] Laing R. O. (1979). Three cases of poisoning by Boophane disticha. Central Afr. J. Med. 25 (12), 265–266. 544032

[B28] Mamadalieva N. Z. (2012). Phytoecdysteroids from Silene plants: distribution, diversity and biological (antitumour, antibacterial and antioxidant) activities. Bol. Latinoam. del Caribe Plantas Med. Aromáticas 11 (6), 474–497.

[B29] Manganyi M. C. Bezuidenhout C. C. Regnier T. Ateba C. N. (2021). A chewable cure “kanna”: biological and pharmaceutical properties of Sceletium tortuosum. Molecules 26 (9), 2557. 10.3390/molecules26092557 33924742 PMC8124331

[B30] Maroyi A. (2019). A synthesis and review of medicinal uses, phytochemistry and biological activities of Helichrysum odoratissimum (L) sweet. Asian J. Pharm. Clin. Res. 12, 15–23. 10.22159/ajpcr.2019.v12i18.33508

[B31] Maroyi A. (2022). Medicinal uses, phytochemistry and pharmacological properties of Dioscorea dregeana (Kunth) T. Durand & Schinz. Med. Plants-International J. Phytomedicines Relat. Industries 14 (1), 57–63. 10.5958/0975-6892.2022.00006.5

[B32] Matsushima Y. Eguchi F. Kikukawa T. Matsuda T. (2009). Historical overview of psychoactive mushrooms. Inflamm. Regen. 29 (1), 47–58. 10.2492/inflammregen.29.47

[B33] Mhame P. P. Busia K. Kasilo O. M. J. (2014). Clinical practices of African traditional medicine. Afr. Health Monitor-Special Issue 14, 32–39.

[B34] Mitchell P. Hudson A. (2004). Psychoactive plants and southern African hunter-gatherers: a review of the evidence. South. Afr. Humanit. 16 (1), 39–57.

[B35] Mondolot L. La Fisca P. Buatois B. Talansier E. De Kochko A. Campa C. (2006). Evolution in caffeoylquinic acid content and histolocalization during Coffea canephora leaf development. Ann. Bot. 98 (1), 33–40. 10.1093/aob/mcl080 16675605 PMC2803532

[B36] Morgan-Trimmer S. Wood F. (2016). Ethnographic methods for process evaluations of complex health behaviour interventions. Trials 17 (1), 1–11. 10.1186/s13063-016-1340-2 27142662 PMC4855482

[B37] Mouhamed Y. Vishnyakov A. Qorri B. Sambi M. Frank S. S. Nowierski C. (2018). Therapeutic potential of medicinal marijuana: an educational primer for health care professionals. Drug, Healthc. patient Saf. 10, 45–66. 10.2147/DHPS.S158592 29928146 PMC6001746

[B38] Naidoo D. Roy A. Slavětínská L. P. Chukwujekwu J. C. Gupta S. Van Staden J. (2020). New role for crinamine as a potent, safe and selective inhibitor of human monoamine oxidase B: *in vitro* and *in silico* pharmacology and modeling. J. Ethnopharmacol. 248, 112305. 10.1016/j.jep.2019.112305 31639490

[B39] Nair J. J. van Staden J. (2014). Cytotoxicity studies of lycorine alkaloids of the Amaryllidaceae. Nat. Product. Commun. 9 (8), 1934578X1400900–1210. 10.1177/1934578x1400900834 25233606

[B40] Nsuala B. N. Enslin G. Viljoen A. (2015). “Wild cannabis”: a review of the traditional use and phytochemistry of Leonotis leonurus. J. Ethnopharmacol. 174, 520–539. 10.1016/j.jep.2015.08.013 26292023

[B41] Nyazema N. Z. (1984). Poisoning due to traditional remedies. Central Afr. J. Med. 30 (5), 80–83. 6467363

[B42] Olatunji T. L. Siebert F. Adetunji A. E. Harvey B. H. Gericke J. Hamman J. H. (2021). Sceletium tortuosum: a review on its phytochemistry, pharmacokinetics, biological and clinical activities. J. Ethnopharmacol. 280, 114476. 10.1016/j.jep.2021.114476 34333104

[B43] Orr C. Spechler P. Cao Z. Albaugh M. Chaarani B. Mackey S. (2019). Grey matter volume differences associated with extremely low levels of cannabis use in adolescence. J. Neurosci. 39 (10), 1817–1827. 10.1523/JNEUROSCI.3375-17.2018 30643026 PMC6407302

[B44] Osemwegie O. Okhuoya J. Dania T. (2014). Ethnomycological conspectus of West African mushrooms: an awareness document. Adv. Microbiol. 4, 39–54. 10.4236/aim.2014.41008

[B45] Papadopulos J. Mentis A.-F. A. Liapi C. (2021). Social pharmacology as an underappreciated field in medical education: a single medical school’s experience. Front. Pharmacol. 12, 714707. 10.3389/fphar.2021.714707 34531746 PMC8438604

[B46] Pasquali L. (2021). Leshoma, the visionary plant of southern Africa. Antrocom Online J. Anthropol. 17 (1).

[B47] Patel D. M. Galani V. J. (2017). Evaluation of neuropharmacological activity of Dioscorea bulbifera using various experimental models. Adv. Plants Agric. Res. 7 (1), 00241. 10.15406/apar.2017.07.00241

[B48] Rätsch C. (2005). The encyclopedia of psychoactive plants: ethnopharmacology and its applications. Simon & Schuster.

[B49] Rispel L. Setswe G. (2007). Stewardship: protecting the public's health: oversight: principles and Policy. South Afr. health Rev. 2007 (1), 3–17.

[B50] Roth G. (2004). The quest to find consciousness. Sci. Am. Mind 14 (1), 32–39. 10.2307/24939363

[B51] Russo E. B. (2017). Cannabidiol claims and misconceptions. Trends Pharmacol. Sci. 38 (3), 198–201. 10.1016/j.tips.2016.12.004 28089139

[B52] Serabele K. Chen W. Combrinck S. (2023). “Helichrysum odoratissimum,” in The South African herbal pharmacopoeia (Massachusetts, United States: Academic Press), 247–258.

[B53] Sobiecki J. F. (2008). A review of plants used in divination in southern Africa and their psychoactive effects. South. Afr. Humanit. 20 (2), 333–351. 10520/EJC84811

[B54] Soni P. Siddiqui A. A. Dwivedi J. Soni V. (2012). Pharmacological properties of Datura stramonium L. as a potential medicinal tree: an overview. Asian Pac. J. Trop. Biomed. 2 (12), 1002–1008. 10.1016/S2221-1691(13)60014-3 23593583 PMC3621465

[B55] Sparg S. Light M. E. Van Staden J. (2004). Biological activities and distribution of plant saponins. J. Ethnopharmacol. 94 (2-3), 219–243. 10.1016/j.jep.2004.05.016 15325725

[B56] Stafford G. I. Pedersen M. E. van Staden J. Jäger A. K. (2008). Review on plants with CNS-effects used in traditional South African medicine against mental diseases. J. Ethnopharmacol. 119 (3), 513–537. 10.1016/j.jep.2008.08.010 18775771

[B57] Stamets P. (1996). Psilocybin mushrooms of the world: an identification guide. Berkeley, CA: Speed Press, 10.

[B58] Sumpter L. (2021). A closer look at the dream enhancing Silene capensis. Available at: https://helathfoodinnovationmanagement.academia.edu/louovcm (Accessed July 21, 2023).

[B59] Tundis R. Statti G. A. Conforti F. Bianchi A. Agrimonti C. Sacchetti G. (2005). Influence of environmental factors on composition of volatile constituents and biological activity of Helichrysum italicum (Roth) Don (Asteraceae). Nat. Prod. Res. 19 (4), 379–387. 10.1080/1478641042000261969 15938146

[B60] University of San Francisco, UCSF (2023). UCSF psilocybin clinical trials - San Francisco Bay Area. Available at: https://clinicaltrials.ucsf.edu/psilocybin.

[B61] van der Horst R. (2018). Observation 311217: Psilocybe cubensis (earle) singer. Available at: https://mushroomsobserver.org/observation/311217.

[B62] Viljoen A. Chen W. Nduvho M. Kamatou G. Sandasi M. (2022). “Helichrysum odoratissimum,” in Phytochemical profiling of commercially import South African plants. eBook chapter 11. ISBN 9780128237809.

[B63] WHO (2023). Drugs (psychoactive) who.int/health-topics/drugs-psychoactive.

[B64] Zandkarimi F. Decatur J. Casali J. Gordon T. Skibola C. Nuckolls C. (2023). Comparison of the cannabinoid and terpene profiles in commercial cannabis from natural and artificial cultivation. Molecules 28 (2), 833. 10.3390/molecules28020833 36677891 PMC9861703

[B65] Zibareva L. Yeriomina V. I. Munkhjargal N. Girault J. P. Dinan L. Lafont R. (2009). The phytoecdysteroid profiles of 7 species of Silene (Caryophyllaceae). Archives Insect Biochem. Physiology Publ. Collab. Entomological Soc. Am. 72 (4), 234–248. 10.1002/arch.20331 19750548

[B66] Ziff S. Stern B. Lewis G. Majeed M. Gorantla V. R. (2022). Analysis of psilocybin-assisted therapy in medicine: a narrative review. Cureus 14 (2), e21944. 10.7759/cureus.21944 35273885 PMC8901083

