# Supplementary figures and images for: The use and potential abuse of psychoactive plants in southern Africa: an overview of evidence and future potential
Source: Front Pharmacol. 2024 May 24;15:1269247. doi: 10.3389/fphar.2024.1269247 (PMC11162113; doi:10.3389/fphar.2024.1269247)

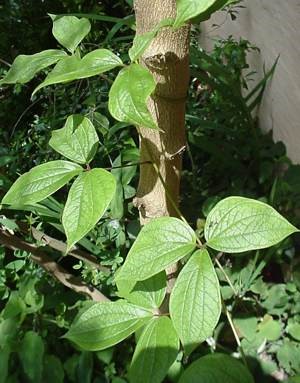

Supplement: Supplementary file 1 [file Image9.JPEG]

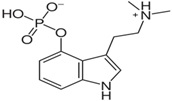

Supplement: Supplementary file 2 [file Image1.JPEG]

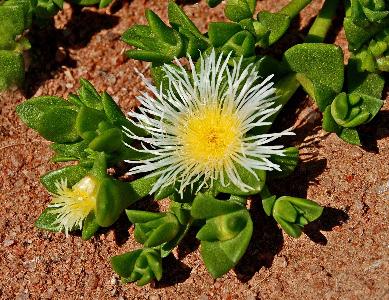

Supplement: Supplementary file 3 [file Image4.JPEG]

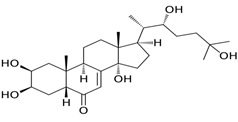

Supplement: Supplementary file 4 [file Image7.JPEG]

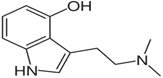

Supplement: Supplementary file 5 [file Image2.JPEG]

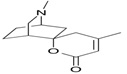

Supplement: Supplementary file 6 [file Image10.JPEG]

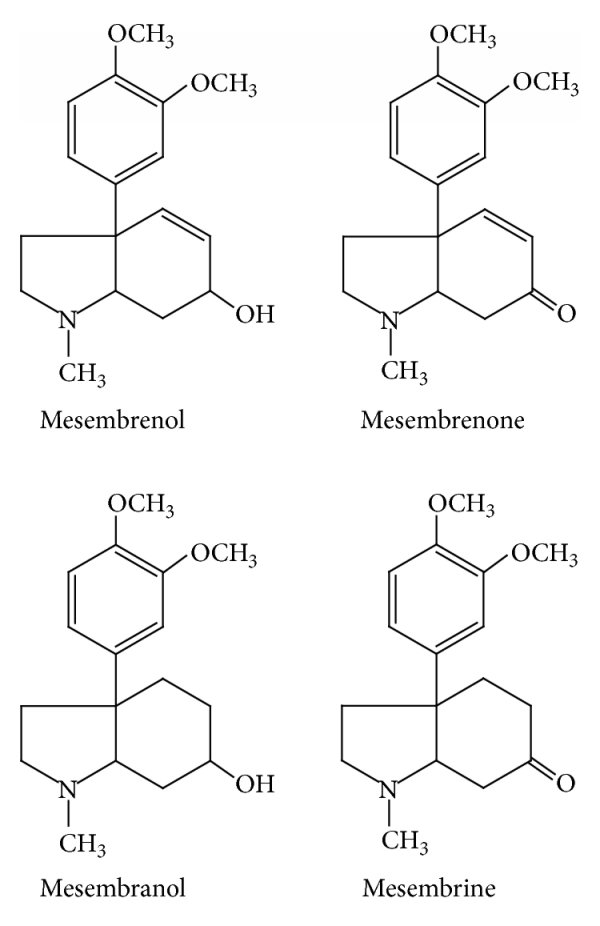

Supplement: Supplementary file 7 [file Image5.PNG]

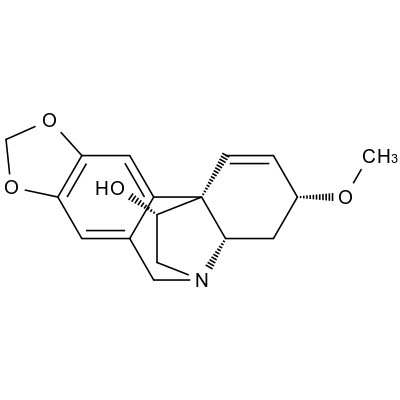

Supplement: Supplementary file 8 [file Image11.JPEG]

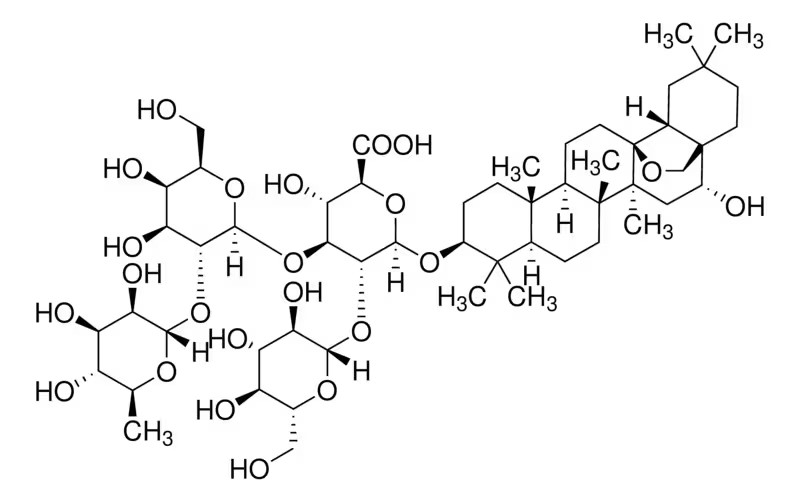

Supplement: Supplementary file 9 [file Image8.JPEG]

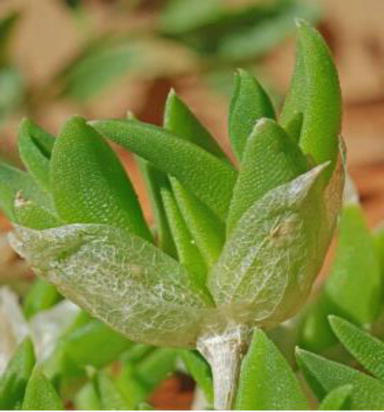

Supplement: Supplementary file 10 [file Image3.PNG]

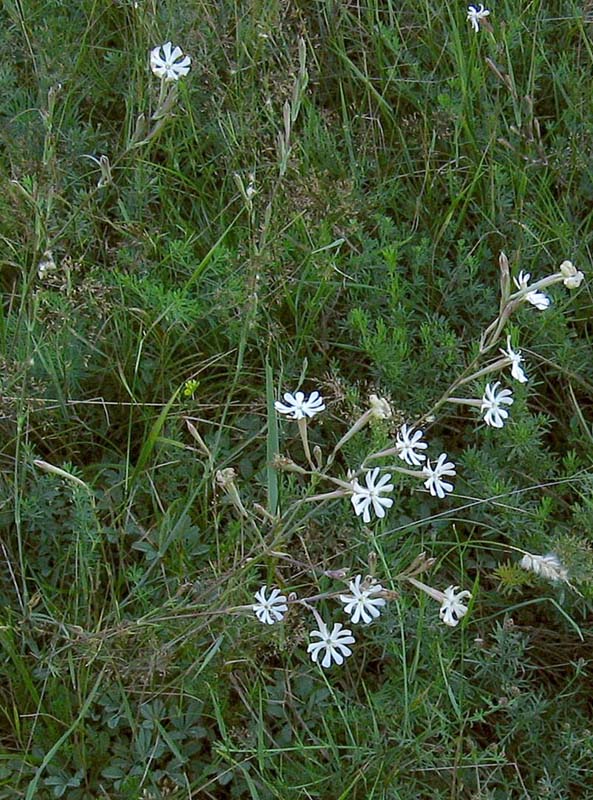

Supplement: Supplementary file 11 [file Image6.JPEG]
